# Resistance makes us stronger: What can Uncle Toni teach us about medical training?

**DOI:** 10.3389/fsurg.2022.964505

**Published:** 2022-08-03

**Authors:** Philipp Taussky

**Affiliations:** Department of Neurosurgery, Clinical Neurosciences Center, University of Utah, Salt Lake City, UT, United States

**Keywords:** training, neurosurgery, education, active learning & teaching methodologies, sports & exercise medicine

As a surgeon and a teacher of medical students, residents, and fellows, I have recently been thinking a lot about Rafael Nadal and his exploits at the 2022 French Open Tournament. My thoughts are not strictly medical, related to his many injuries. Instead, I ponder how a “geriatric” 36-year-old tennis player can perform at a level that exceeds that of much younger men. The question may be unexpected coming from a surgeon. But surgery is a high-performance specialty, and surgeons are constantly looking to improve their own performance. Tennis, with its emphasis on performing with consistency at the highest level with a low tolerance for “unforced errors,” may provide valuable insights.

Until recently, the common belief was that tennis after the age of 30 becomes a declining affair. The demands of the game are just too high to be able to perform at the highest athletic level, particularly after many years on the professional circuit and the related wear and tear on the aging body. As a result, it is surprising that the current top players are all in their thirties, and the next generation of players have not been able to replace them at the top of the sport. Although we look in awe at Djokovic's (35 years), Serena Williams's (40 years), and Rafael Nadal's (36 years) ability to stay at the top of the professional rankings, we also wonder why the young players, reared on modern performance analysis by a multi-disciplinary and comprehensive approach that includes nutritionists, sports psychologists, video analysis, trainers, and physical therapists, do not surpass them.

The fact that the next generation of tennis players have not been able to replace the elders of tennis has not been lost on players, coaches, commentators, and lovers of the sport. The point was recently discussed by Toni Nadal, the uncle and life-long coach (until recently) of Rafael Nadal, in a TED talk to a Spanish audience. Uncle Toni, as he is commonly known, is considered the most successful tennis coach of all times, with 17 Grand Slam tournament titles with Rafael. Uncle Toni is also well known for his tough training methods in which his nephew Rafael had to play on bad courts with bad balls and no water in an effort to prepare him for the hardships for the professional tour. In one anecdote, Rafael played a juniors match with a broken racket and lost the first set 6–0. His uncle asked him afterwards why, as a tennis player with his experience, he did not notice his racket was broken. Rafael answered that he was so used to being at fault for everything, it had never crossed his mind that his poor performance might be due to a broken racket. But Toni's approach to training was not callous or cruel; it was part of a wider philosophy that was meant to force Rafael to deal with adversities, adapt to them, and avoid the easy trap of finding excuses and blaming extraneous factors for poor performance. His training, no doubt, was a far cry from current academies that support the players with high-tech solutions. Thus, Uncle Toni proposes a reason for the continued superiority of the older players: The young players have lost sight of the forest for the trees. In their high-tech quest for performance enhancement, they have lost sight of what it takes to be successful in tennis: endurance, determination, insight, grit, drive, and an excitement for the game.

It is a simple answer to a complex question, but Toni Nadal's observations about professional tennis may also ring true for medicine. In a hyper-modernized medical world, with an emphasis on making our professional life more convenient by the use of digital imaging, electronic medical records, wellness programs, mid-level providers, and work-hour restrictions for residents, our young trainees are in danger of losing sight of the most important aspects of our discipline, or as Toni Nadal would say, its essence. For physicians, this involves our devotion to our patients; life-long self-study of surgical anatomy, physiology, and pathology; an appreciation of our colleagues who share our work load and our values; a commitment to reduce pain and suffering; and an appreciation of our mentorship and patronage training system.

And we wonder, what if Uncle Toni is right? By eliminating the hardships of training, have we robbed our trainees of the opportunity to develop a philosophical mindset that emphasizes personal responsibility, encourages insight, avoids finding excuses and blaming others, and fosters a focus on performance. Trainees are a bit like family. Neurosurgery residency training takes 7 years, or about the average length of an American marriage, so we develop very close ties. No one wants to see ones' children suffer, and we all appreciate the fact that our trainees in many ways have it better than previous generations. But the question remains, what if by eliminating the mundane hardships of the trainees' lives—sometimes referred to as “scut work”—we are actually setting them up for failure? What if our efforts as educators should not be channeled towards making the trainees’ lives easier and more convenient but should focus on the essence of our surgical values. The essence of surgical performance is not so dissimilar to tennis; it takes determination, insight, drive, grit, and a true devotion to our patients.

Modern technology may free us to spend our time more efficiently, but it will not replace our core values. Our trainees, with more time on their hands, need to be guided to remember the true essence of our surgical being: to alleviate suffering. How much the trainees have to suffer in order to get there, we don't know, but according to Uncle Toni, it may be a lot. And with Rafael Nadal winning a record 22 Grand Slams, Uncle Toni may just be right.

